# Successful Retreatment With ADOC Chemotherapy in Relapsed Thymic Carcinoma: Experiences in Two Cases

**DOI:** 10.4021/wjon435w

**Published:** 2012-02-19

**Authors:** Yayoi Nomura, Tomonobu Koizumi, Akihiro Kitaguchi, Toshimichi Horiuchi, Shintarou Kanda, Hiroshi Yamamoto, Masayuki Hanaoka, Keishi Kubo

**Affiliations:** a First Department of Internal Medicine, Shinshu University School of Internal Medicine, 3-1-1, Asahi Matsumoto, Japan

**Keywords:** Thymic cancer, Sensitive relapse, Chemotherapy

## Abstract

The optimal second-line chemotherapeutic regimen for thymic carcinoma remains uncertain and predictive factors for the response have not been identified. We encountered two cases of relapsed thymic carcinoma with recurrence 1.5 and 8 years after initial response to cisplatin/doxorubicin/vincristine/cyclophosphamide (ADOC) chemotherapy. Both cases were successfully retreated with ADOC. Our observations suggest that relapsed thymic carcinoma occurring a long treatment-free time from the initial response may be sensitive to the previous chemotherapy. We described two cases of relapsed thymic carcinoma successfully retreated with ADOC chemotherapy. Both patients had partial response to initial ADOC and long disease free times.

## Introduction

Thymic carcinoma is a thymic epithelial neoplasm with cytological malignant features and a clinical course that tends to be much more aggressive than that of thymoma [1]. Thymic carcinoma also tends to metastasize widely, which leads to a highly lethal course [1-3]. Thus, the role of systemic chemotherapy may be important in the treatment of inoperable thymic carcinoma. Cisplatin-based chemotherapy as the first-line chemotherapy has repeatedly been shown to benefit certain patients [1-6]. However, an optimal regimen and the role of second-line therapy remain unclear. Here, we describe two cases of thymic carcinoma showing good response to initial cisplatin/doxorubicin/vincristine/cyclophosphamide (ADOC) chemotherapy with relapse after long intervals. Readministration of ADOC resulted in good outcome in both cases. We describe the clinical impacts of these two cases that have provided new insight into chemotherapy-sensitive relapse in thymic carcinoma.

## Case Report

### Case 1

**Figure 1:**
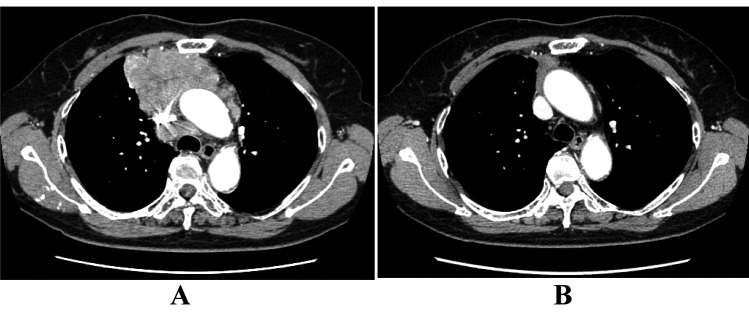
The response to initial ADOC chemotherapy in case 1. Chest computed tomography before initial ADOC chemotherapy (A) and after four cycles of initial ADOC chemotherapy (B).

**Figure 2:**
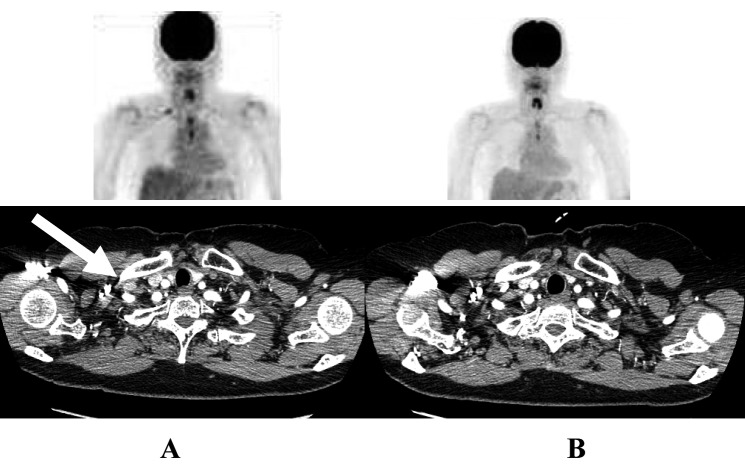
The response to second-line ADOC chemotherapy in case 1. Chest computed tomography and positron emission tomography before initial ADOC chemotherapy (A) and after four cycles of second line ADOC chemotherapy (B).

A 70-year-old woman was referred to our hospital in October 2007 because of chest discomfort and anterior mediastinal mass on chest computed tomography (CT) (Fig. 1A). Percutaneous CT-guided biopsy was performed and the lesions were confirmed histologically to be epidermoid-type thymic carcinoma. She was treated with 4 cycles of a combination of cisplatin (50 mg/m^2^) and doxorubicin (40 mg/m^2^) on day 1, vincristine (0.6 mg/m^2^) on day 3, and cyclophosphamide (700 mg/m^2^) on day 4 (ADOC chemotherapy), and showed partial response to the treatment (Fig. 1B). Subsequently, the residual tumor was surgically resected in March 2008, followed by a total of 60 Gy (2 Gy × 30 days) thoracic radiotherapy. In October 2009, positron emission tomography (PET) and chest CT scan revealed right clavicular lymph node swelling (Fig. 2A). Although biopsy was not performed, this was considered relapsed thymic cancer. She was retreated with modified ADOC chemotherapy using nedaplatin (80 mg/m^2^) instead of cisplatin because of her reduced renal function (creatinine clearance < 50 ml/min). After 4 cycles of modified ADOC, the patient showed a complete response (Fig. 2B). As of October 2011, the patient is still alive without any symptoms or further recurrence.

### Case 2

**Figure 3:**
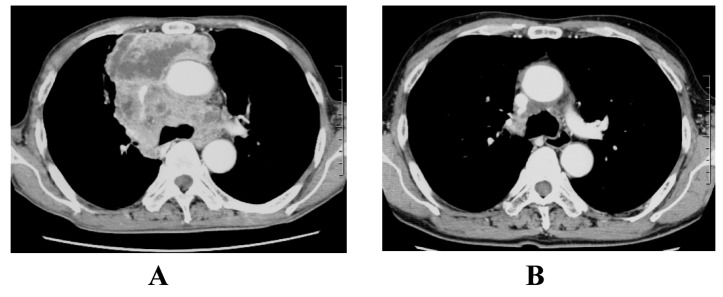
The response to initial ADOC chemotherapy in case 2. Chest computed tomography before initial ADOC chemotherapy (A) and after five cycles of initial ADOC chemotherapy (B).

**Figure 4:**
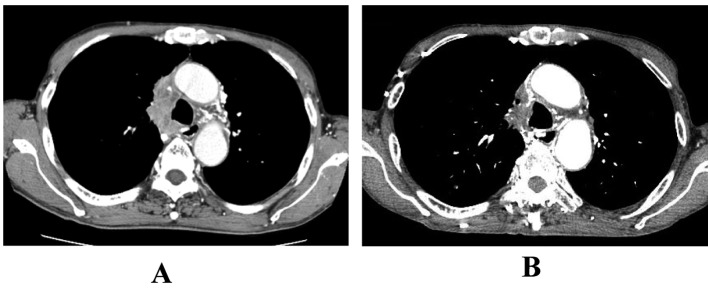
The response to second-line ADOC chemotherapy in case 2. Chest computed tomography before second-line ADOC chemotherapy (A) and after a cycle of second-line ADOC chemotherapy (B).

A 65-year-old man was admitted to our hospital because of face edema, general fatigue and appetite loss in July 2002. Chest X-ray and chest CT scan revealed an anterior mediastinal mass (Fig. 3A). After histological diagnosis of thymic carcinoma (squamous cell carcinoma) by percutaneous CT-guided biopsy, he was treated with 5 cycles of ADOC chemotherapy. Good partial response was observed (Fig. 3B). In April 2011, he presented supra vena cava syndrome again. CT scan indicated a mediastinal mass (Fig. 4A). Bronchoscopic examination was performed and transbronchial needle biopsy revealed recurrence of thymic cancer. Readministration of ADOC was initiated in August 2011. After one cycle of ADOC chemotherapy, chest CT scan revealed a partial response (Fig. 4B). However, the patient developed febrile neutropenia and bacterial pneumonia during the first cycle of chemotherapy. He refused subsequent chemotherapy and is currently under observation without symptoms.

## Discussion

We reported two patients with relapsed thymic carcinoma who were successfully retreated with ADOC chemotherapy. Both patients responded well to the initial ADOC chemotherapy and developed recurrence after a long interval. Our experience suggested that thymic carcinoma is chemosensitive and that the same regimen may be useful as an alternative second-line chemotherapy regimen in certain patients with advanced thymic carcinoma.

Chemotherapy is commonly employed in patients with unresectable and/or metastatic thymic carcinoma [1-6]. Yoh et al. [4] evaluated the efficacy of CODE (cisplatin, vincristine, doxorubicin, and etoposide) therapy for thymic carcinoma and reported a response rate of 42%. Recently, we evaluated 34 patients with unresectable thymic carcinoma treated with ADOC chemotherapy and described a 50% response rate and median survival time of 21.3 months [5]. These findings suggest that thymic carcinoma is a relatively chemosensitive type of tumor. However, the majority of patients showed relapse and require second-line chemotherapy. Several cases of advanced thymic carcinoma treated with salvage chemotherapies have been reported [7, 8], but the role and optimal chemotherapy for second-line chemotherapy remain unclear.

In general, two major factors have been proposed to predict the efficacy of salvage chemotherapy in malignancies, in particular chemosensitive tumors [9-13], i.e., the response to initial chemotherapy and the duration of treatment-free time (TFT) between last exposure to chemotherapy and recurrence [9-13]. Relapsed patients with a longer TFT could be considered to be sensitive, and those with a shorter TFT to be refractory to the efficacy of subsequent chemotherapy, although TFT is dependent on the tumor type [9-13]. Repeated use of the original induction regimen is recommended as the most common form of treatment for sensitive relapsed patients, because of the favorable clinical outcomes [9-13]. Our cases of thymic carcinoma showed a good response to initial ADOC and had long TFT over several years, consistent with the findings of “sensitive relapse” patients with other malignancies [9-13]. Although the optimal TFT in thymic carcinoma for distinguishing sensitive from refractory relapse remains undetermined, our experience provides new insight for the proper selection for chemotherapy regimens for relapsed thymic carcinoma.

Performance status at the time of recurrence is also important in both efficacy of second-line chemotherapy and survival after therapy [14]. The physical conditions of both of the present cases were good before second-line chemotherapy. One patient developed neutropenic fever and pneumonia during the first cycle of ADOC readministration, resulting in deteriorated performance status (PS) of 2. However, he has subsequently been well with no symptoms (PS 0).

In summary, our cases of relapsed thymic carcinoma suggested that readministration of the same regimen should be considered as the second-line chemotherapy in patients who responded to initial therapy and have a long treatment-free time. In addition, our observations emphasize that thymic carcinoma is a chemotherapy-sensitive type of tumor.
